# Surgeon peer network characteristics and adoption of new imaging techniques in breast cancer: A study of perioperative MRI

**DOI:** 10.1002/cam4.1821

**Published:** 2018-11-15

**Authors:** Sara S. Tannenbaum, Pamela R. Soulos, Jeph Herrin, Craig E. Pollack, Xiao Xu, Nicholas A. Christakis, Howard P. Forman, James B. Yu, Brigid K. Killelea, Shi‐Yi Wang, Cary P. Gross

**Affiliations:** ^1^ Yale University School of Medicine New Haven Connecticut; ^2^ Cancer Outcomes, Public Policy and Effectiveness Research (COPPER) Center Yale Cancer Center and Yale School of Medicine New Haven Connecticut; ^3^ Section of General Internal Medicine, Department of Internal Medicine Yale School of Medicine New Haven Connecticut; ^4^ Section of Cardiology, Department of Internal Medicine Yale School of Medicine New Haven Connecticut; ^5^ Health Research & Educational Trust Chicago Illinois; ^6^ Johns Hopkins School of Medicine Baltimore Maryland; ^7^ Johns Hopkins Bloomberg School of Public Health Baltimore Maryland; ^8^ Department of Obstetrics, Gynecology and Reproductive Sciences Yale School of Medicine New Haven Connecticut; ^9^ Department of Sociology Yale University New Haven Connecticut; ^10^ Yale Institute for Network Science and Human Nature Lab, Yale University New Haven Connecticut; ^11^ Department of Radiology and Biomedical Imaging Yale School of Medicine New Haven Connecticut; ^12^ Department of Therapeutic Radiology Yale School of Medicine New Haven Connecticut; ^13^ Department of Surgery Yale School of Medicine New Haven Connecticut; ^14^ Yale Cancer Center New Haven Connecticut; ^15^ Department of Chronic Disease Epidemiology Yale School of Public Health New Haven Connecticut

**Keywords:** breast cancer, diagnostic imaging, magnetic resonance imaging, Medicare, social networking

## Abstract

**Background:**

Perioperative MRI has disseminated into breast cancer practice despite equivocal evidence. We used a novel social network approach to assess the relationship between the characteristics of surgeons’ patient‐sharing networks and subsequent use of MRI.

**Methods:**

We identified a cohort of female patients with stage 0‐III breast cancer from the Surveillance, Epidemiology, and End Results (SEER)‐Medicare database. We used claims data from these patients and non‐cancer patients from the 5% Medicare sample to identify peer groups of physicians who shared patients during 2004‐2006 (T1). We used a multivariable hierarchical model to identify peer group characteristics associated with uptake of MRI in T2 (2007‐2009) by surgeons who had not used MRI in T1.

**Results:**

Our T1 sample included 15 149 patients with breast cancer, treated by 2439 surgeons in 390 physician groups. During T1, 9.1% of patients received an MRI; the use of MRI varied from 0% to 100% (IQR 0%, 8.5%) across peer groups. After adjusting for clinical characteristics, patients treated by surgeons in groups with a higher proportion of primary care physicians (PCPs) in T1 were less likely to receive MRI in T2 (OR = 0.81 for 10% increase in PCPs, 95% CI = 0.71, 0.93). Surgeon transitivity (ie, clustering of surgeons) was significantly associated with MRI receipt (*P *= 0.013); patients whose surgeons were in groups with higher transitivity in T1 were more likely to receive MRI in T2 (OR = 1.29 for 10% increase in clustering, 95% CI = 1.06, 1.58).

**Conclusion:**

The characteristics of a surgeon's peer network are associated with their patients’ subsequent receipt of perioperative MRI.

## 1 INTRODUCTION

Perioperative MRI has been adopted into clinical practice despite equivocal results about effectiveness. Earlier evidence suggested that MRI may increase detection of occult cancer,^1^, ^2^ but subsequent studies have shown that perioperative MRI has generally not yielded better outcomes, may be associated with more aggressive surgical approaches, and can lead to overdiagnosis.^3^, ^4^, ^5^, ^6^, ^7^, ^8^ Additionally, women who receive MRIs in the diagnostic or perioperative setting have higher total, imaging and biopsy costs.^9^ Despite these concerns, perioperative MRI use increased from <1% of Medicare beneficiaries undergoing breasts cancer surgery in 2000‐2001, to about 25% of patients in 2008‐2009.^10^

Understanding why physicians use unproven technologies, such as perioperative MRI, is an important step to improving value in cancer care. New technologies represent a major contributor to the cost increase in cancer care.^9^, ^11^ Previous work has demonstrated the influence of several factors, including patient preference, payer policies, and geographic region, on variation in physician practices.^12^, ^13^, ^14^ Although these are important determinants of practice patterns, these prior studies have not focused on the potential impact that physicians have on each other. Indeed, one novel factor that has emerged as a potentially important influence on physicians’ practices is their peer network. Social contagion theory proposes that social networks can have a measurable impact on health‐related behaviors and traits^15^ such as smoking and obesity.^16^, ^17^ More recently, studies of physician peer networks have shown differences in care patterns and outcomes based on peer connections.^18^, ^19^, ^20^ For example, sharing patients with another surgeon who had incorporated brachytherapy into breast cancer care was associated with surgeons’ subsequent uptake of brachytherapy.^18^ Similarly, physician peer group use of perioperative MRI was associated with subsequent adoption of the technology.^21^

Despite these initial studies suggesting that social contagion may affect cancer care, we know little about whether specific characteristics of physician peer groups might be related to the adoption of new treatments. Several studies have shown that physician peer networks are complex and can vary in both their composition^22^ and connectivity.^23^ There is also evidence that these characteristics are associated with differences in care. Physician peer groups with a higher proportion of primary care physicians (PCPs) experienced higher rates of ambulatory care sensitive admissions.^22^ Additionally, network connectivity features including centrality (the extent to which connections between other providers in the network go through that provider) and clustering (whether the neighbors of each physician in a network are more or less connected with each other) have been associated with differences in care.^24^, ^25^ For example, increased centrality of PCPs was associated with fewer medical specialist visits.^24^

To our knowledge, no study has addressed whether specific network characteristics are associated with the uptake of specific medical practices over time. Identifying the factors which affect the social structure of medical care could help shed new light on potential mechanisms of social contagion, and allow for proactive identification of physician networks at higher risk of adopting low‐value practices. To address this knowledge gap, we constructed peer groups of physicians who treated Medicare beneficiaries with breast cancer and examined how characteristics varied across these physician networks. We then tested whether those characteristics were associated with differences in rates of adoption of perioperative MRI over time.

## 2 METHODS

### 2.1 Study design

We used a retrospective claims‐based approach to construct physician networks within hospital referral regions (HRRs) during a period of rapid uptake of MRI (2004‐2009). We identified a cohort of Medicare beneficiaries with stage 0‐III breast cancer from the Surveillance, Epidemiology and End Results (SEER)‐Medicare database during 2004‐2006 (T1) and a corresponding cohort of non‐cancer patients with physician visits during the same time period. The claims from these patients were used to map connections between five types of physicians involved in breast cancer care (surgeons, medical oncologists, radiologists, radiation oncologists, and PCPs including obstetricians/gynecologists). Next, we characterized these physician peer groups using metrics adapted from the field of social network analysis, which we hypothesized might be associated with adoption of a new technology such as use of perioperative MRI in breast cancer care. We then identified surgeons who had not used MRI during T1 (2004‐2006) and examined whether physician peer group characteristics were associated with subsequent adoption of perioperative MRI use during T2 (2007‐2009) among these surgeons. The approach is similar to what we used previously to show that the rate of MRI use in a physician peer group was associated with adoption of MRI in a subsequent time period.^21^

### 2.2 Data source and sample

Our study used the SEER‐Medicare database, which contains clinical and demographic information about cancer patients and connects this information with a patient's Medicare claims. The SEER regions cover 28% of the United States population.^26^

We used two different samples for this study. First, in order to identify physician patient‐sharing networks, we identified female beneficiaries who were diagnosed with stage 0‐III breast cancer during T1. We excluded patients who had an additional cancer diagnosis, were outside the age range of 67‐94, were not continuously enrolled in Medicare Parts A and B for 12 months before and after diagnosis, had the diagnosis reported only on autopsy or death certificate, had an unknown diagnosis month, or had non‐epithelial histology. To increase the amount of observed patient‐sharing between physicians, we also used Medicare claims for women from the Medicare 5% random sample of beneficiaries residing in SEER regions who did not have cancer. We assigned these women without cancer a random index date, which was used in an analogous way as date of diagnosis for the beneficiaries with breast cancer.

Then, to assess the impact of peer group characteristics on the adoption of MRI, we selected women with breast cancer who met the same eligibility criteria described above and were diagnosed during T2, and who were treated by surgeons who had no MRI use in T1. In addition, we further excluded women from this sample who did not receive cancer surgery and those with in situ cancer.

### 2.3 Physician peer group construction

Our method of physician peer group construction has been described previously elsewhere.^21^ Physicians were included in peer group construction if they were PCPs, radiologists, or cancer specialists (medical oncologists, surgeons, radiation oncologists) and provided care for five or more patients (cancer or non‐cancer) in the sample during the T1 study period. We identified the specialty of each physician using the Medicare specialty code as well as billings for certain services (eg, breast surgery for surgeons). Patient‐sharing ties were determined by identifying all National Provider Index physician identifier codes on a patient's claims during the 3 months before through 9 months after diagnosis. A connection between physicians was established if they shared at least two patients.

**Figure 1:**
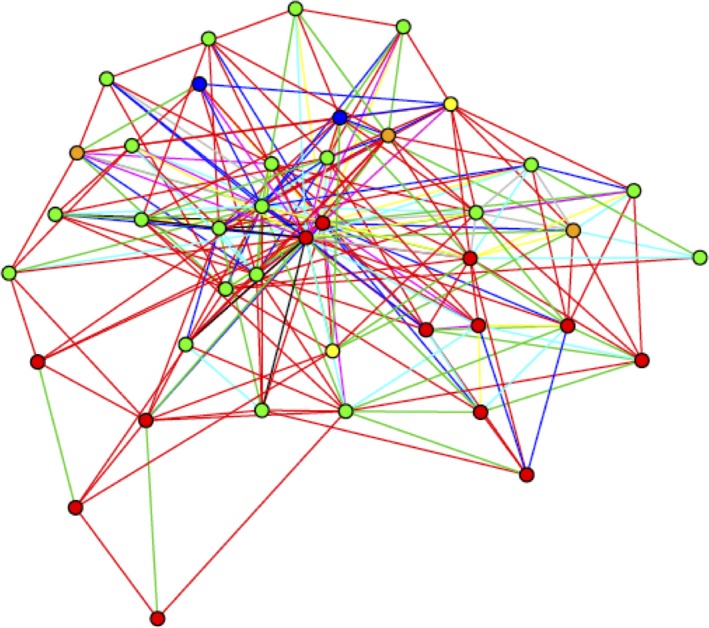
Peer group example

After mapping patient‐sharing connections between physicians using all patients residing within an HRR, we constructed mutually exclusive physicians within each HRR using the Girvan‐Newman algorithm, which identifies an optimal number of discrete highly connected sub‐groups within the larger HRR‐based networks. We weighted each connection between two physicians by the number of patients shared.^27^, ^28^ Once these physician peer groups were constructed, each breast cancer patient was assigned to the surgeon who performed her breast cancer surgery and then subsequently assigned to the physician peer group to which her surgeon was assigned. In our analysis, we excluded physician peer groups that contained only one surgeon or one breast cancer patient. An example of a physician peer group is shown in Figure 1.

### 2.4 Physician peer group characteristics

For each physician peer group, we calculated the following characteristics:

#### 2.4.1 Composition

This included measures for the number of women with breast cancer assigned to the physician peer group, the total number of patients seen by doctors in that peer group, and the number of physicians by specialty.

#### 2.4.2 Degree

The degree of a physician is the number of other physicians (“alters”) with whom a particular physician of interest (the “ego”) shares patients. As the number of connections with other physicians depends on the number of patients a physician treats, we adjusted the degree by dividing by the total number of patients in a physician peer group. We then calculated the average degree and average adjusted degree for physicians within each physician peer group.

#### 2.4.3 Transitivity

**Figure 2:**
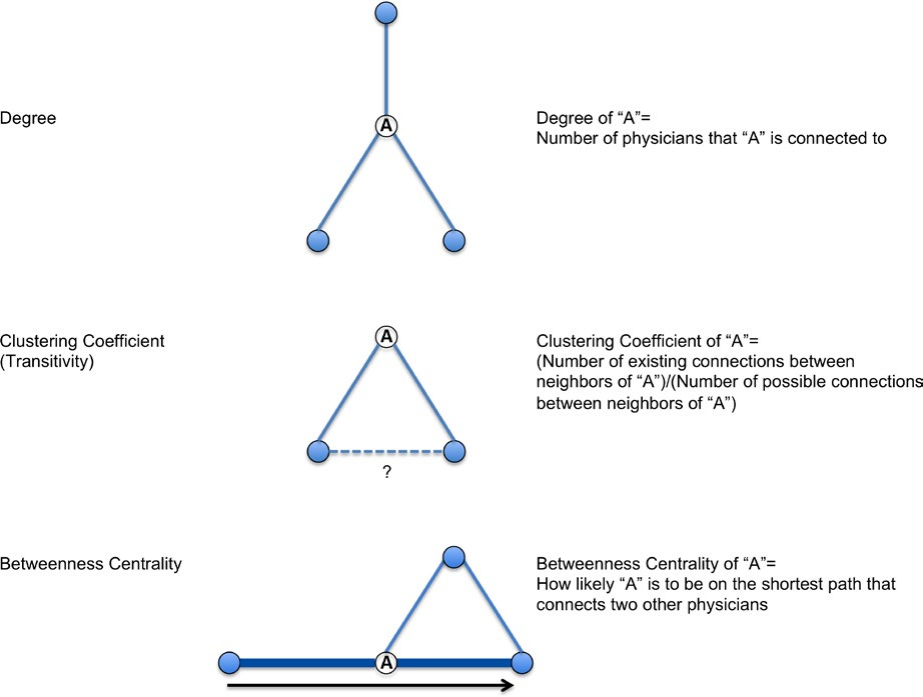
Graphic representation of network terms

Transitivity, measured by a clustering coefficient, reflects how tightly knit a physician's neighbors are (Figure 2). To calculate the clustering coefficient of physician A, we divided the actual connections between neighbors of A by the possible connections between neighbors of A. We used this coefficient for each physician to calculate the average clustering coefficient for each physician specialty in the physician peer group. We also calculated the global clustering coefficient, which is the percentage of completed triangles over all potential triangles of physicians in the peer group. In addition, we calculated network density (observed connections in a group over all possible connections).

#### 2.4.4 Centrality

Centrality is a measure of physician importance in their peer group (Figure 2). We calculated “betweenness centrality” which reflects how likely a given physician is to be on the shortest path that connects two other physicians within their peer group.^23^ To calculate the centrality by physician specialty, we took the average centrality of each specialty and divided it by the overall mean centrality of all physicians in the peer group.

#### 2.4.5 Outcome and covariates

Our outcome was whether a patient with breast cancer had a claim for perioperative MRI imaging (identified by CPT/HCPCS codes). Perioperative MRI was defined as an MRI received three months prior to diagnosis to three months following surgery.

Patient demographic and clinical characteristics included age at diagnosis, race, number of comorbidities, whether they had a PCP visits in the 24 through 3 months prior to diagnosis, marital status, median household income at the census tract or zip code level. Comorbidity was measured using a modified Elixhauser index from 12 to 1 month prior to breast cancer diagnosis.^29^ Additionally, we measured cancer characteristics including tumor size, node positivity, stage, hormone receptor status, grade, and laterality.

### 2.5 Statistical analysis

We summarized each of the physician peer group characteristics described above, reporting the mean, standard deviation, median, interquartile range (IQR) and range. If a physician peer group did not include a particular specialty, its metrics for that specialty were not reported. Then, to assess the association between each characteristic and the uptake of MRI during T2, we estimated a series of hierarchical generalized linear models, with a logistic link and random effects for HRR, physician peer group, and physician. All models included all the patient characteristics listed above as well as the baseline (T1) peer group rate of MRI. We first estimated a separate model for each peer group characteristic, and tested whether the effect of the characteristic differed from zero, and reported the effect, the *P*‐value, and the proportion of variance explained at the peer group level.

For all physician peer group characteristics that were significant at *P* < 0.05 level in the individual assessment, we then used a collinear diagnostic to identify characteristics that were highly correlated with one another based on the method of Belsley and Kuh to identify and drop highly collinear factors.^30^ Following the collinear diagnostic, all remaining physician peer group characteristics were included in a multivariable model (with adjustment for patient characteristics and peer group rate of T1 MRI use, as well as HRR, peer group, and physician random effects). We further applied backward stepwise selection to this model to determine the final list of variables retained.

All analyses were done using SAS 9.4, R (version 3.2.2) (SAS Institute Inc., Cary, NC), the igraph package (v 0.9) (R Foundation for Statistical Computing, Vienna, Austria), and Stata 14.2 (StataCorp 2016, College Station TX).

## 3 RESULTS

At T1, after the exclusion of physician peer groups with <2 physicians, there were 15 149 breast cancer patients across 390 peer groups in 91 HRRs (characteristics of T1 patients are shown in Appendix Table S1). There were 2439 surgeons, 1214 radiation oncologists, 1532 medical oncologists, 5890 radiologists, and 11 638 PCPs. During T1, peer group MRI use varied from 0% to 100% (IQR 0%, 8.5%), with an overall baseline MRI use of 9.1%.

There were 1117 surgeons who had no MRI patients in T1 and also treated patients in T2. The characteristics of patients in our T2 sample treated by these surgeons are described in Table 1. Over half of our T2 patients were stage I (58.3%), and the majority of patients were hormone receptor positive (82.0%).

**Table 1:** Characteristics of T2 patients (2007‐2009)

Characteristic	N (%)
Age	
66‐69	3047 (22.7)
70‐74	3334 (24.9)
75‐79	3055 (22.8)
80‐84	2375 (17.7)
85‐94	1595 (11.9)
Race	
White	11 987 (89.4)
Black	915 (6.8)
Other	504 (3.8)
Elixhauser group	
No conditions	7150 (53.3)
1‐2 conditions	4845 (36.1)
3+ conditions	1411 (10.5)
PCP visit	
No	857 (6.4)
Yes	12 549 (93.6)
Marital status	
Married	5963 (44.5)
Unmarried	6969 (52.0)
Unknown	474 (3.5)
Income category	
Q1	2612 (19.5)
Q2	2003 (14.9)
Q3	2870 (21.4)
Q4	2687 (20.0)
Q5	3232 (24.1)
Tumor size	
<2.0 cm	8502 (63.4)
2‐5 cm	4251 (31.7)
>5 cm	570 (4.3)
Missing	83 (0.6)
Node status	
No/Unknown	10 245 (76.4)
Yes	3161 (23.6)
Cancer stage	
Stage I	7822 (58.3)
Stage II	4331 (32.3)
Stage III	1253 (9.3)
Receptor status	
None	1809 (13.5)
Estrogen or Progesterone	10 995 (82.0)
Missing	602 (4.5)
Cancer grade	
1	3488 (26.0)
2	5909 (44.1)
3	3378 (25.2)
4	72 (0.5)
Missing	559 (4.2)
Tumor laterality	
Right‐sided	6623 (49.4)
Left‐sided	6781 (50.6)

PCP, primary care physicians

### 3.1 Physician peer group characteristics

At T1, there was a mean of 4.7 (SD = 3.6) physician peer groups within each HRR. Peer groups had an average of 38.8 (SD = 46.4) surgeries, 58.2 (SD = 61.0) physicians (Table 2), and 372.4 (SD = 388.4) cancer and non‐cancer patients. PCPs were the most common specialty, accounting for 47.7% (SD = 17.9) of the physicians within a peer group on average. Surgeons accounted for an average of 13.5% (SD = 9.4) of the physicians, and medical oncologists and radiation oncologists each made up a small percentage, accounting for 6.2% (SD = 5.2) and 5.8% (SD = 8.6) on average, respectively.

**Table 2:** Peer group characteristics in T1 (2004‐2006)

Peer group characteristic	Number of peer groups	Mean (SD)
Number of surgeries	390	38.8 (46.4)
Number of physicians	390	58.2 (61.0)
% of PCP	390	47.7 (17.9)
% cancer patients/all patients	390	4.3 (4.8)
Degree	390	14.8 (8.6)
Adjusted degree	390	0.843 (0.336)
Observed/max. connections	390	0.349 (0.147)
Average physician centrality	390	24.9 (35.3)
PCP Centrality	363[Link]	0.080 (0.097)
Surgeon centrality	371[Link]	0.142 (0.152)
Average physician transitivity	381[Link]	0.589 (0.142)
PCP transitivity	371[Link]	0.828 (0.091)
Surgeon transitivity	380[Link]	0.769 (0.110)

PCP, primary care physicians.

Degree = Number of other physicians with whom the “ego” shares patients.

Centrality = How likely the “ego” is to be on the shortest path between two other physicians.

Transitivity (Clustering Coefficient) = Actual number of connections between neighbors of the “ego” divided by possible number of connections between neighbors of the “ego”.

Some peer groups did not have enough physicians in that specialty to calculate this characteristic.

Network characteristics related to position of different specialties and connectivity varied widely between networks. The average overall physician centrality was 24.9 (SD = 35.3). The relative centrality by specialty was highest for radiologists (0.52, SD = 0.21) and lowest for PCPs (0.080, SD = 0.097). The centrality for surgeons was 0.14 (SD = 0.15). Looking at the average number of connections, the mean physician degree was 14.8 (SD = 8.6), indicating that the average physician shared patients with about 15 other physicians. The mean adjusted degree was 0.84 (SD = 0.34). Average surgeon clustering coefficients (a measure of transitivity) varied across physician peer groups from 0.41 to 1.

### 3.2 Association between physician peer group characteristics and MRI adoption

Several physician peer group characteristics were significantly associated with adoption of MRI during T2 when examined individually, and these included number of PCPs, number of surgeons, radiologist transitivity, number of patients, percent surgeons and others listed in Table 3. Of those significant in the individual analysis, three were dropped from consideration for the final model because of collinearity (number of patients, percent surgeons, and average centrality).

**Table 3:** Association between peer group characteristics and MRI adoption in T2 (2007‐2009) analyses

Peer group characteristic	Individual variable analysis		Final model	
	Odds ratio (95% CI)	*P*‐value	Odds ratio (95% CI)	*P*‐value
Number of surgeries	1.00 (0.99, 1.00)	0.02		
Number of physicians	1.00 (0.99, 1.00)	0.02		
10% increase in PCPs	0.81 (0.73, 0.89)	0.001	0.81 (0.71, 0.93)	0.003
% cancer patients/all patients	1.02 (1.01, 1.04)	0.01		
Degree	0.99 (0.97, 1.01)	0.21		
Adjusted degree	0.84 (0.49, 1.44)	0.52		
Observed/max connections	1.74 (0.50, 6.02)	0.38		
Average physician centrality	1.00 (0.99, 1.00)	0.02		
PCP centrality	2.28 (0.21, 25.24)	0.50		
Surgeon centrality	0.39 (0.07, 2.24)	0.29		
10% increase average physician transitivity	1.26 (1.08, 1.49)	0.005		
10% increase PCP transitivity	1.10 (0.87, 1.40)	0.43		
10% increase surgeon transitivity	1.39 (1.14, 1.69)	0.001	1.29 (1.06, 1.58)	0.01

CI, confidence interval; MRI, magnetic resonance imaging; PCP, primary care physicians.

Both analyses adjusted for the following covariates: age, race, Elixhauser comorbidity, PCP visits, marital status, income, tumor size, node positive, stage, receptor, grade, laterality, and peer group level T1 MRI use.

Two physician peer group characteristics, the percentage of PCPs and surgeon transitivity, were significantly associated with uptake of MRI in the final model, which also adjusted for patient characteristics and baseline physician peer group MRI use. About 10% higher surgeon transitivity was associated with higher rates of MRI adoption (odds ratio [OR] = 1.29, 95% confidence interval [CI] = 1.06, 1.58). Conversely higher percentage of PCPs was associated with lower rates of MRI adoption; each 10% increase in the percentage of PCPs was associated with an OR of 0.81 (95% CI = 0.71, 0.93). In addition to the two peer group structural characteristics, we found that peer group baseline rates of MRI use were also associated with adoption of MRI; patients treated by physicians who were in groups with >10% baseline MRI use were significantly more likely to receive MRI during T2 (OR: 4.11; 95% CI = 2.42, 6.97) compared to groups with no MRI use during T1.

## 4 DISCUSSION

We found that two physician peer group characteristics were associated with surgeon adoption of breast MRI in a longitudinal analysis. A higher concentration of PCPs in the peer group was associated with decreased adoption, while increased surgeon transitivity was associated with increased adoption. While prior work suggests that membership in patient‐sharing groups is associated with differences in physician behavior,^19^, ^20^, ^21^, ^22^, ^24^ our findings further suggest that the composition and structure of physician peer groups can impact uptake of a technology. Our study builds upon prior work by incorporating a longitudinal design, allowing us to focus on adoption of a new modality.

A higher percentage of PCPs within a peer group was associated with lower rates of MRI adoption. This result adds to the limited, cross‐sectional, and conflicting evidence about the role of PCP position in networks and utilization of healthcare resources. A previous study found that networks with a higher PCP percentage had slightly higher ambulatory care sensitive hospital admissions.^22^ Additionally, in our study, we did not find a significant association with PCP centrality, while a previous study found that a higher relative PCP centrality was associated with lower resource utilization and costs in the last two years of life.^24^

Since PCPs do not order MRIs, it is not immediately clear why a physician network with more PCPs would have a slower adoption of MRI. A previous study has shown an association of the co‐localization of surgical care and PCPs at the same hospital with significantly lower costs of care in colon cancer, suggesting that PCP peer relationships and placement in care structures may impact resource utilization in cancer care.^31^ However, evidence on the relationship between PCP concentration and spending is mixed. One study showed that states with a higher percentage of general practitioners had lower spending and improvement in quality of care metrics,^32^ whereas a later study showed higher spending in areas with more PCP full‐time equivalents per Medicare beneficiary.^33^ The percentage of PCPs could also be a proxy for other health system characteristics that affect surgeon usage of MRI, such as strategic goals emphasizing value‐based care. Further research is needed to elucidate our observed negative association between PCP concentration and MRI adoption.

Increased transitivity, as we studied in surgeons, can allow for people to receive similar information from multiple sources, allowing for reinforcement of a practice that may not be readily adopted from exposure to a single source of information.^34^ In a study where subjects were assigned to a random or clustered network, the clustered network spread the health behavior (registering for a health forum) farther and faster.^35^ Thus, surgeons within highly clustered networks may have had the reinforcement necessary to overcome the activation energy of adopting a new technology.

Our study has limitations. First, patient‐sharing connections identified based on billing patterns may not represent true relationships between physicians. However, prior work demonstrated that physicians who share patients on billing claims are more likely to report a professional relationship.^36^ Second, despite being limited to one mutually exclusive peer group within an HRR, doctors could be assigned to an additional peer group in different HRRs. Third, our sample was limited to Medicare fee for service patients. Fourth, despite controlling for a rich set of demographic and clinical covariates, unmeasured confounders may still exist, including physician factors, such as surgeon's experience. Lastly, our study did not examine how other healthcare delivery structures, such as hospital affiliations, may have impacted our results. Previous work has shown that peer groups may be associated with, but are distinct from, these structures.^23^

In conclusion, we expanded on prior work by incorporating a longitudinal study design and showed that the structure of physician peer groups may influence the adoption of new technologies. These structures could be exploited for interventions aiming to improve value by reducing usage of ineffective techniques. Further research is needed to better characterize the mechanisms by which care patterns spread across peer groups, and assess whether these findings are generalizable to the use of other technologies in cancer care.

## DISCLOSURES

Dr Wang receives research support from Genentech. The source of support was not used for any portion of the current manuscript. Dr Yu is a consultant for Augmenix Inc Dr Pollack has stock ownership in Gilead Pharmaceuticals. Dr Killelea is a consultant for Genentech. Drs. Gross and Yu and Ms Soulos receive research funding from 21st Century Oncology LLC. Dr Gross has also received funding from Medtronic and Johnson & Johnson. Ms Tannenbaum and Drs. Herrin, Christakis, Forman, and Xu report no relationships or activities that could appear to have influenced the submitted work.

## References

[1] Bilimoria KY , Cambic A , Hansen NM , et al. Evaluating the impact of preoperative breast magnetic resonance imaging on the surgical management of newly diagnosed breast cancers. Arch Surg. 2007;142(5):441‐445; discussion 445‐447. 17515485 10.1001/archsurg.142.5.441

[2] Fischer U , Zachariae O , Baum F , et al. The influence of preoperative MRI of the breasts on recurrence rate in patients with breast cancer. Eur Radiol. 2004;14(10):1725‐1731. 15248080 10.1007/s00330-004-2351-z

[3] Weber JJ , Bellin LS , Milbourn DE , et al. Selective preoperative magnetic resonance imaging in women with breast cancer: no reduction in the reoperation rate. Arch Surg. 2012;147(9):834‐839. 22987175 10.1001/archsurg.2012.1660

[4] Young P , Kim B , Malin JL . Preoperative breast MRI in early‐stage breast cancer. Breast Cancer Res Treat. 2012;135(3):907‐912. 22923237 10.1007/s10549-012-2207-1

[5] Houssami N , Turner R , Macaskill P , et al. An individual person data meta‐analysis of preoperative magnetic resonance imaging and breast cancer recurrence. J Clin Oncol. 2014;32(5):392‐401. 24395846 10.1200/JCO.2013.52.7515

[6] Houssami N , Turner R , Morrow M . Preoperative magnetic resonance imaging in breast cancer: meta‐analysis of surgical outcomes. Ann Surg. 2013;257(2):249‐255. 23187751 10.1097/SLA.0b013e31827a8d17

[7] Turnbull L , Brown S , Harvey I , et al. Comparative effectiveness of MRI in breast cancer (COMICE) trial: a randomised controlled trial. Lancet. 2010;375(9714):563‐571. 20159292 10.1016/S0140-6736(09)62070-5

[8] Wang SY , Long JB , Killelea BK , et al. Preoperative breast magnetic resonance imaging and contralateral breast cancer occurrence among older women with breast cancer. J Clin Oncol. 2016;34(4):321‐328. 26628465 10.1200/JCO.2015.62.9741 PMC4872032

[9] Onega T , Tosteson AN , Weiss J , et al. Costs of diagnostic and preoperative workup with and without breast MRI in older women with a breast cancer diagnosis. BMC Health Serv Res. 2016;16:76. 26920552 10.1186/s12913-016-1317-6 PMC4769533

[10] Killelea BK , Long JB , Chagpar AB , et al. Trends and clinical implications of preoperative breast MRI in Medicare beneficiaries with breast cancer. Breast Cancer Res Treat. 2013;141(1):155‐163. 23942872 10.1007/s10549-013-2656-1 PMC3893026

[11] Roberts KB , Soulos PR , Herrin J , et al. The adoption of new adjuvant radiation therapy modalities among Medicare beneficiaries with breast cancer: clinical correlates and cost implications. Int J Radiat Oncol Biol Phys. 2013;85(5):1186‐1192. 23182396 10.1016/j.ijrobp.2012.10.009 PMC3606652

[12] Matlock DD , Groeneveld PW , Sidney S , et al. Geographic variation in cardiovascular procedure use among Medicare fee‐for‐service vs Medicare Advantage beneficiaries. JAMA. 2013;310(2):155‐162. 23839749 10.1001/jama.2013.7837 PMC4021020

[13] So C , Kirby KA , Mehta K , et al. Medical center characteristics associated with PSA screening in elderly veterans with limited life expectancy. J Gen Intern Med. 2012;27(6):653‐660. 22180196 10.1007/s11606-011-1945-9 PMC3358397

[14] Krein SL , Hofer TP , Kerr EA , et al. Whom should we profile? Examining diabetes care practice variation among primary care providers, provider groups, and health care facilities. Health Serv Res. 2002;37(5):1159‐1180. 12479491 10.1111/1475-6773.01102 PMC1464024

[15] Christakis NA , Fowler JH . Social contagion theory: examining dynamic social networks and human behavior. Stat Med. 2013;32(4): 556‐577. 22711416 10.1002/sim.5408 PMC3830455

[16] Christakis NA , Fowler JH . The spread of obesity in a large social network over 32 years. N Engl J Med. 2007;357(4):370‐379. 17652652 10.1056/NEJMsa066082

[17] Christakis NA , Fowler JH . The collective dynamics of smoking in a large social network. N Engl J Med. 2008;358(21):2249‐2258. 18499567 10.1056/NEJMsa0706154 PMC2822344

[18] Pollack CE , Soulos PR , Gross CP . Physician's peer exposure and the adoption of a new cancer treatment modality. Cancer. 2015;121(16):2799‐2807. 25903304 10.1002/cncr.29409 PMC4529814

[19] Pollack CE , Wang H , Bekelman JE , et al. Physician social networks and variation in rates of complications following prostatectomy. Value Health. 2014;17(5):611‐618. 25128055 10.1016/j.jval.2014.04.011 PMC4135395

[20] Pollack CE , Weissman G , Bekelman J , et al. Physician social networks and variation in prostate cancer treatment in three cities. Health Serv Res. 2012;47(1 Pt 2):380‐403. 22092259 10.1111/j.1475-6773.2011.01331.x PMC3258347

[21] Pollack CE , Soulos PR , Herrin J , et al. The impact of social contagion on physician adoption of advanced imaging tests in breast cancer. J Natl Cancer Inst. 2017;109(8):djw330. 10.1093/jnci/djw330 PMC6059114 28376191

[22] Casalino LP , Pesko MF , Ryan AM , et al. Physician networks and ambulatory care‐sensitive admissions. Med Care. 2015;53(6):534‐541. 25906013 10.1097/MLR.0000000000000365

[23] Landon BE , Keating NL , Barnett ML , et al. Variation in patient‐sharing networks of physicians across the United States. JAMA. 2012;308(3):265‐273. 22797644 10.1001/jama.2012.7615 PMC3528342

[24] Barnett ML , Christakis NA , O’Malley AJ , et al. Physician patient‐sharing networks and the cost and intensity of care in US hospitals. Med Care. 2012;50(2):152‐160. 22249922 10.1097/MLR.0b013e31822dcef7 PMC3260449

[25] Hollingsworth JM , Funk RJ , Garrison SA , et al. Differences between physician social networks for cardiac surgery serving communities with high versus low proportions of black residents. Med Care. 2015;53(2):160‐167. 25517071 10.1097/MLR.0000000000000291

[26] National Cancer Institute . Overview of the SEER Program. https://seer.cancer.gov/about/overview.html. Accessed January 13, 2017.

[27] Newman ME . Modularity and community structure in networks. Proc Natl Acad Sci USA. 2006;103(23):8577‐8582. 16723398 10.1073/pnas.0601602103 PMC1482622

[28] Girvan M , Newman ME . Community structure in social and biological networks. Proc Natl Acad Sci USA. 2002;99(12):7821‐7826. 12060727 10.1073/pnas.122653799 PMC122977

[29] Elixhauser A , Steiner C , Harris DR , et al. Comorbidity measures for use with administrative data. Med Care. 1998;36(1):8‐27. 9431328 10.1097/00005650-199801000-00004

[30] Belsley DA , Kuh E , Welsch RE . Regression Diagnostics: Identifying Influential Data and Sources of Collinearity. Hoboken, NJ: Wiley; 2013.

[31] Hussain T , Chang H‐Y , Luu N‐P , et al. The value of continuity between primary care and surgical care in colon cancer. PLoS ONE. 2016;11(5):e0155789. 27219454 10.1371/journal.pone.0155789 PMC4878733

[32] Baicker K , Chandra A . Medicare spending, the physician workforce, and beneficiaries' quality of care. Health Aff (Millwood). 2004;Suppl Web Exclusives:W4–184‐197. 10.1377/hlthaff.w4.184 15451981

[33] Chang C , Stukel TA , Flood A , et al. Primary care physician workforce and medicare beneficiaries' health outcomes. JAMA. 2011;305(20):2096‐2104. 21610242 10.1001/jama.2011.665 PMC3108147

[34] Centola D , Macy M . Complex contagions and the weakness of long ties. Am J Sociol. 2007;113(3):702‐734.

[35] Centola D . The spread of behavior in an online social network experiment. Science. 2010;329(5996):1194‐1197. 20813952 10.1126/science.1185231

[36] Barnett ML , Landon BE , O'Malley AJ , et al. Mapping physician networks with self‐reported and administrative data. Health Serv Res. 2011;46(5):1592‐1609. 21521213 10.1111/j.1475-6773.2011.01262.x PMC3207194

