# Supplementary material for: Surgeon peer network characteristics and adoption of new imaging techniques in breast cancer: A study of perioperative MRI
Source: Cancer Med. 2018 Nov 15;7(12):5901–9. doi: 10.1002/cam4.1821 (PMC6308117; doi:10.1002/cam4.1821)
Supplement: Supplementary file 1 [file CAM4-7-5901-s001.docx]

Table S1: Characteristics of T1 patients (2004-2006)

| **Characteristic** | | **N (%)** |
| --- | --- | --- |
|  |  |  |
| Age |  |  |
|  | 66-69 | 3254 (20.8) |
|  | 70-74 | 3849 (24.6) |
|  | 75-79 | 3806 (24.3) |
|  | 80-84 | 2955 (18.9) |
|  | 85-94 | 1788 (11.4) |
|  |  |  |
| Race |  |  |
|  | White | 14136 (90.3) |
|  | Black | 1024 (6.5) |
|  | Other | 492 (3.1) |
|  |  |  |
| Elixhauser Group |  |  |
|  | No conditions | 8759 (56.0) |
|  | 1-2 conditions | 5468 (34.9) |
|  | 3+ conditions | 1425 (9.1) |
|  |  |  |
| PCP visit |  |  |
|  | No | 1065 (6.8) |
|  | Yes | 14587 (93.2) |
|  |  |  |
| Marital Status |  |  |
|  | Married | 6829 (43.6) |
|  | Unmarried | 8264 (52.8) |
|  | Unknown | 559 (3.6) |
|  |  |  |
| Income Category |  |  |
|  | Q1 | 3211 (20.5) |
|  | Q2 | 2367 (15.1) |
|  | Q3 | 3352 (21.4) |
|  | Q4 | 3147 (20.1) |
|  | Q5 | 3573 (22.8) |
|  |  |  |
| Tumor Size |  |  |
|  | <2.0cm | 9688 (61.9) |
|  | 2cm-5cm | 5090 (32.5) |
|  | >5cm | 739 (4.7) |
|  | Missing | 135 (0.9) |
|  |  |  |

Appendix Table 1 Continued: Characteristics of T1 patients (2004-2006)

| Node Status |  |  |
| --- | --- | --- |
|  | No/Unknown | 11682 (74.6) |
|  | Yes | 3970 (25.4) |
|  |  |  |
| Cancer Stage |  |  |
|  | Stage I | 8894 (56.8) |
|  | Stage II | 5131 (32.8) |
|  | Stage III | 1627 (10.4) |
|  |  |  |
| Receptor Status |  |  |
|  | None | 2169 (13.9) |
|  | Estrogen or Progesterone | 12401 (79.2) |
|  | Missing | 1082 (6.9) |
|  |  |  |
| Cancer Grade |  |  |
|  | 1 | 3754 (24.0) |
|  | 2 | 6789 (43.4) |
|  | 3 | 4037 (25.8) |
|  | 4 | 177 (1.1) |
|  | Missing | 895 (5.7) |
|  |  |  |
| Tumor Laterality |  |  |
|  | Right-sided | 7684 (49.1) |
|  | Left-sided | 7966 (50.9) |
